# Comprehensive allergy work-up is mandatory in cystic fibrosis patients who report a history suggestive of drug allergy to beta-lactam antibiotics

**DOI:** 10.1186/2045-7022-2-10

**Published:** 2012-06-14

**Authors:** Silvia Caimmi, Céline Sanfiorenzo, Davide Caimmi, Philippe-Jean Bousquet, Raphael Chiron, Pascal Demoly

**Affiliations:** 1 Allergy unit, Deépartement de Pneumologie et Addictologie, Hôpital Arnaud-de-Villeneuve, University Hospital of Montpellier and Inserm U657, 37, avenue du Doyen Gaston Giraud, 34295, Montpellier cedex 5, France; 2 Centres de Ressources et de Compétences de la Mucoviscidose (CRCM), Montpellier, France

**Keywords:** DAHD, Beta-lactam, Drug allergy, Cystic fibrosis

## Abstract

**Background:**

In the general population, reports on suspected ß-lactam hypersensitivity are common. After a drug allergy work-up at best 20% of the selected patients are positive. However, these considerations have not been explored in cystic fibrosis patients for whom antibiotics are even more crucial.

**Methods:**

The study, part of the Drug Allergy and Hypersensitivity (DAHD) cohort, was performed in the regional cystic fibrosis center of Montpellier, France. After identifying patients with a clinical history suggestive of drug allergy to ß-lactams, a complete drug allergy work-up, was carried out according to the EAACI recommendations.

**Results:**

Among the 171 patients involved, 23 reported clinical manifestations potentially compatible with a drug allergy to ß-lactams. After performing the complete drug-allergy work-up, 7 were considered as drug hypersensitive (3 had positive skin tests, 1 a positive provocation test, 3 declined the tests). Excluding the latter 3 patients with incomplete drug allergy work-up, the rate of proven drug allergy was 2.3%.

**Conclusions:**

Drug allergy to ß-lactams in cystic fibrosis patients is of importance. A full drug allergy work-up is mandatory in case of suspicion, because ß-lactam responsibility is often ruled out.

## Background

Cystic fibrosis (CF) is a chronic disease for which chronic therapies are required to slow the progression of disease [1]. Patients with CF suffer daily symptoms, frequent exacerbations of pulmonary infection, and an early demise [2]. Chronic airway inflammation and infection are indeed the greatest causes of mortality and morbidity in patients with CF, the resulting lung damages being the main cause of death [1,3,4]. Antibiotics are typically used for early, intermittent infection, with the goal being to eradicate the pathogen [2]. Antibacterial therapies are instituted empirically and are individualized based on both patient’s factors (severity of exacerbation, frequency of exacerbation, recent courses of anti-infectives) and pathogen factors (previously isolated pathogens and *in vitro* predicted antibiotic susceptibilities) [5].

The French registry on CF reported that, in 2007, more than 75% of the 4806 patients who underwent a common cytobacteriologic examination of the sputum (93.5% of the CF patients treated in France) were infected by a non-commensal bacteria (mainly *Staphylococcus aureus* and *Pseudomonas aeruginosa*) and, amongst them, 1837 (35.7%) had received at least one course of antibiotics during the previous year [6].

The frequent use of antibiotics in patients presenting with CF is often associated to hypersensitivity clinical manifestations, commonly leading to patients being considered allergic to one or more antibiotics, and therefore such drugs being contra-indicate. Thus, the prevalence of drug allergy is reported to be three times greater (6 to 22%) than the one observed in the general population [7-9]. However, as for the general population, it could be suspected that in many cases the clinical manifestation was misunderstood, and falsely considered as a drug allergy [10,11].

In the general population, allergy to betalactams (BLs) is the most frequent cause of drug reaction, mediated by specific immunological mechanisms [12]. Such reactions may be induced by all BLs currently available [12]. Such an assumption has to be true even in patients with CF, who are much more exposed to these molecules than the general population. The allergy work-up involves careful history-taking followed by a drug provocation test (DPT) when skin tests are negative [12]. If skin tests and/or DPT are positive, a different BL is often found as an alternative.

The present study aimed to assess the prevalence of patients presenting clinical manifestations considered as a drug allergy to BLs, one of the most commonly used antibiotics in CF patients. Moreover, the study also looked for the prevalence of proven drug allergy after a thorough drug allergy work-up.

## Methods

### Population

The study was performed over two years (between 2009 and 2011) in the regional CF center of Montpellier, south of France. This center aimed to manage all patients presenting with a CF in the Languedoc-Roussillon region and treated according to the international EFS recommendations. Local ethical committee approved the study design and protocols. The study was part of the historical-prospective cohort study Drug Allergy and Hypersensitivity Database (DAHD). After informing and receiving the written informed consent from the patients, or from each parent of minors, the medical referee was asked to identify and contact those with a clinical history suggestive of drug allergy to a BL (Table 1).

**Table 1:** Characteristics of patients with a suspected hypersensibility to beta-lactams

**Patients with compatible history of B-lactams hypersensitivity**	23	
Males	16	(69.6%)
Mean age at diagnosis	8.5	(SD 13.3)
Mean age at reaction	21.2	(SD 11.2)
Patients' Genotype (CFTR gene)		
*F508del/F508del*	15	(65.2%)
*F508del/R1162 X*	1	(4.3%)
*F508del/N1303 K*	1	(4.3%)
*F508del/R553 X*	1	(4.3%)
*F508del/R1102 X*	1	(4.3%)
*F508del/3272-2 G > A*	1	(4.3%)
*Other/other*	3	(13.0%)
Bacterium at first reaction		
*Pseudomonas aer.*	11	(47.8%)
*Staph. aureus*	4	(17.4%)
*Other*	2	(8.7%)
*Unknown*	6	(26.1%)

### History

For patient with a suspicion of allergy to BLs, an allergist trained in drug allergy filled in the standardized ENDA (European Network for Drug Allergy) questionnaire on drug allergy [13]. Patients with a suspected hypersensitivity to other classes of antibiotic were not included in the present study. According to the ENDA protocols for immediate [14] and non-immediate [15] reactions, patients with a compatible history of BL allergy underwent a complete allergy work-up (Table 1).

### Skin tests

Skin tests (prick and intradermal) were performed as previously described [16-18] with the classical benzylpenicillin (penicillin G), semi-synthetic penicillins (amoxicillin and ampicillin) and any other suspected BL. Major and minor determinants of penicillin G are not commercialized in France. The procedure ended when a positive skin test was found, according to international guidelines [14,15]. In patients with an unknown chronology or with a non-immediate reaction, a late reading of skin tests was performed [15]. Positive controls for prick tests were carried out with a histamine solution at 10 mg/ml. As a negative control for prick and intradermal tests, normal saline solution was used.

### Drug provocation tests (DPT)

In accordance to the ENDA recommendations [12,14,15], in the case of negative results of skin tests, provocation tests with the suspected BL were performed under strict hospital surveillance [10]. Provocation tests consisted of administering increasing doses of the suspected drug up to the full therapeutic dose or until symptoms of allergy occurred [10]. Administration was performed over a one-day hospitalization, by a physician with full resuscitation back-up. Patients with histories of anaphylactic reactions had intravenous catheters in place during the entire test. Patients who had experienced severe reactions, such as toxic epidermal necrolysis, Stevens-Johnson syndrome, acute generalised exanthematous pustolosis, hypersensitivity syndrome or drug reaction with eosinophilia and systemic symptoms, blood alterations, nephritis, pneumonitis, hepatitis, and vasculitis, were not tested since the provocation test is contra-indicated in such patients [15,19].

Patients had not been taking any antihistamines or other drugs that could affect skin tests or drug provocations. Patients on beta-blockers were requested to ask their cardiologist if they could stop taking the drug for 2 days.

Patient were asked to contact the physician if a reactions occurred in the days following the provocation test in order to identify delayed reactions.

### Statistical analysis

Categorical data were expressed in frequencies and percent, continuous in mean and standard deviation.

## Results

### Patients and reactions

**Figure 1:**
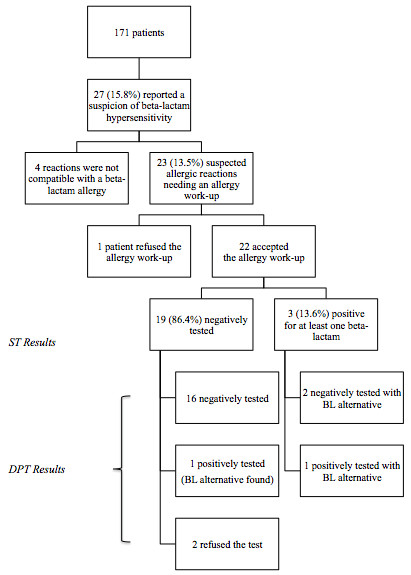
**Flow chart of the present study and of the results.***Legend: BL: Beta-lactam; ST: Skin Tests; DPT: Drug Provocation Test.*

From 1999 to 2009, the regional referent center on CF followed 171 patients, 96 (56%) males, with a mean age at diagnosis of 4 years and a mean age at the time when this study was performed of 18 years. All of them have been exposed to at least one BL course. Among them and during this 10-year period, 27 (15.8%) reported a suspected hypersensitivity reaction to BL antibiotics. Four (14.8%) out of the 27 patients were excluded since the clinical presentation of the reaction was considered as incompatible with a drug allergy by the allergist. Thus, the proportion of patients with a clinical manifestation potentially compatible with a BL allergy was restricted to 23 out of 171, 13.5% (Figure 1). One patient refused to be tested and therefore was excluded from the present study. Twenty-two patients underwent the complete drug allergy work-up. Some patients reported more than one clinical reaction for different ß-lactams, representing a total of 35 reactions. The most common drugs involved were ceftazidime (13 – 37.1% of the overall reactions), followed by piperacillin (10 – 28.6%), and imipenem (5 – 14.3%). The most severe clinical reactions presented by the patients were anaphylaxis/anaphylactic shock (6 – 17.1% of all reactions), followed by urticaria (7 – 20%), and exanthema (4 – 11.4%). Twelve patients (54.5%) presented non-immediate reactions, and 10 (45.5%) immediate reactions (i.e. within the first hour after the last drug intake).

### Skin tests and drug provocation tests

All twenty-two patients underwent skin tests and three were positive, two to one BL only (ticarcillin and piperacillin) and one to two different BLs (ceftazidime and imipenem). All skin reactions appeared in the site of the intradermal test: they occurred at the concentration of 25 mg/ml for piperacillin, ceftazidime and imipenem, and of 2.5 mg/ml for ticarcillin. Two patients with negative skin tests refused the DPT. For twenty patients, at least one DPT was then performed, either with the culprit drug (the 17 remaining negative skin tests patients) or with an alternative when the culprit one was skin test positive. Two patients had a positive DPT, both of them for ceftazidime, one with previous negative skin tests and one with a positive skin test to ticarcillin. The first patient showed a generalized urticaria, 20 minutes after the end of the test (total amount of administered drug: 2,816 grams), while the second one presented a periorbital angioedema, associated with cough right after the administration of a dose of 10 mg of ceftazidime (total amount of administered drug: 16 mg). Three patients presenting multiple histories of suspected drug hypersensitivity to BLs, underwent 2 different DPTs, both of which resulted negative. Therefore, assuming that patients who did not undergo the full drug allergy work-up were sensitized (without any proof other than the clinical presentation and the refusal of the test), 7 patients should be considered as sensitized (4 by drug allergy work-up, 2 who declined the provocation test and 1 who did not undergo the drug allergy work-up). This represented a rate of 4.1% of the total CF cohort or around one third (30.4%) of the patients reporting a clinical history of BL allergy (Figure 1). Excluding the three patients with incomplete drug allergy work-up, the rate of proven drug allergy was 2.3%.

### Follow-up

A 12-months follow-up of the twenty patients was also conducted. One year after the DPT, all patients had at least one new BL antibiotic course. Sixteen patients were re-administered, at least once, the same drug negatively tested. Only one of them re-resented some clinical manifestation after the drug intake, consisting in generalized pruritus and treated by an H1-antihistamine drug, but without stopping the antibiotic course. As for the two patients who resulted to be positive to the DPT, one, for an unknown reason, had a new course of ceftazidime (the drug resulted positive during the DPT) and reacted during the course. Nevertheless, both of them, received, during the 12-months follow-up a BL course with a safe alternative.

## Discussion

The mechanism of acquisition and maintenance of bacterial infection in the airways of patients with CF is unclear [1]. Bacterial growth in biofilms in the CF airway is associated with decreased susceptibility to antibiotics, even when given in combination [1]. The large amount of prescribed antibiotics is correlated with a major risk of developing hypersensitivity reactions. Such a consideration is even more important knowing that the major increase in life expectancy of CF patients is due to a better management of infections [7], and to the wide use of antibiotics [3,4].

This observational historico-prospective study reported 27 (15.8%) suspicions of drug allergy to BLs in 171 CF patients. After performing a full drug-allergy work-up in patients with a suspicion, 4.1% of the patients (30.4% of the patients with a suspicion) were considered as allergic to BL and in 2.3% the allergy was proved by our work-up. These finding suggested that BL drug allergy is not uncommon in CF. However, the rate of sensitized patients could even be overestimated since we considered patients without full drug-allergy work-up as sensitized. The CF specialist in charge of the patient selected all patients who reported for the last 10 years a clinical reaction considered as a BL drug allergy. It is very unlikely that the referring doctor, not specialized in drug allergy, could have missed some patients' reactions, because all adverse events are systematically asked for and listed in the patient files by the CF medical team. Therefore, the initial rate of 15.8% (27 out of 171) patients with a clinical presentation considered as an allergic reaction to BL is a reality. The relatively low rate of “true” sensitized patients, and the good negative predictive value of the BL work-up [20] should encouraged the referring doctor to report the suspicions to a drug allergy specialist.

Some could argue that the present study has a selection bias since it is an observational study focused on a single clinical centre. However, since 2002 the French "Centres de Ressources et de Compétences de la Mucoviscidose" have been created in order to coordinate CF health care, including treatment. These centers cover the whole population, based on the patients’ location. Therefore, such bias should be limited and similar results should be observed for the whole national CF population.

## Conclusions

Drug allergy to BLs in CF patients is of importance since many patients report clinical presentations suggestive of a drug allergy. However, most of these presentations are not a true drug allergy and a full drug allergy work-up is recommended. Otherwise there will be a loss of chance for these patients who often require BLs, and for whom the control of the infection is a major factor of their life expectancy.

## Abbreviations

BL, Beta-lactams; CF, Cystic Fibrosis; DAHD, Drug Allergy and Hypersensitivity Database; DPT, Drug Provocation Test; EAACI, European Academy of Allergy and Clinical Immunology; ENDA, European Network for Drug Allergy.

## Competing interests

The authors declare that they have no competing interests.

## Author’s contributions

SC, CS, and DC performed the drug allergy work-up, collected the data and corrected the manuscript. PJB designed the study, performed the analysis and wrote the manuscript. RC followed the patients, collected the data and corrected the manuscript. PD designed and supervised the study, performed the drug allergy work-up and wrote the manuscript. All authors read and approved the final manuscript.
